# Does erectile dysfunction predict cardiovascular risk? A cross-sectional study of clinical characteristics in patients with erectile dysfunction combined with coronary heart disease

**DOI:** 10.3389/fcvm.2024.1341819

**Published:** 2024-03-18

**Authors:** Luyu Li, Yongtao Zhang, Miaomiao Ma, Feng Liu, Yihan Shang, Quan Yuan, Xiao Li, Baojun Ju

**Affiliations:** ^1^ The First School of Clinical Medicine, The First Clinical Medical College of Henan University of Chinese Medicine, Zhengzhou, Henan, China; ^2^ Department of Andrology, The First Affiliated Hospital of Henan University of Chinese Medicine, Zhengzhou, Henan, China

**Keywords:** erectile dysfunction, cardiovascular disease, coronary heart disease, clinical features, risk factors

## Abstract

**Background:**

Erectile Dysfunction (ED) is a common sexual dysfunction in men who are unable to consistently obtain and maintain sufficient penile erection to accomplish a satisfactory sexual life. ED is currently considered to be a predictor of cardiovascular disease (CVD), but few studies have observed the association between ED and clinical features of coronary heart disease (CHD). An investigation of the association between ED and clinical characteristics of CHD was carried out using a cross-sectional study design.

**Methods:**

This cross-sectional single-center study was conducted in the Department of Cardiology and included 248 patients. Associations between patients' general information, underlying disease information, coronary heart disease information, and ED severity were statistically and analytically analyzed using SPSS 26.0 software. Patients with comparable clinical characteristics were grouped together using K-means clustering. Finally, ordered logistic regression analysis was performed for general and underlying disease information.

**Results:**

In the comparison of general data, age, education, and weekly exercise were associated with the distribution of ED severity. In the comparison of underlying disease information, the number of underlying diseases, hypertension, diabetes, hyperlipidemia, anxiety state, and depressive state were associated with the distribution of ED severity. In the comparison of CHD information, the degree of ED severity was associated with CHD subtypes, lesion sites, number of stenoses, degree of stenosis, and interventional interventions. The time from ED to CHD onset was associated with the subtypes of CHD and the number of stenoses. We clustered the main characteristics of low-risk and high-risk patients and ordered logistic regression analysis found that BMI, smoking, alcoholism, number of underlying diseases, diabetes, anxiety state, and depression state were all risk factors for CHD severity (*P* < 0.05); the higher the value of the above factors, the more severe the degree of CHD. Age was a protective factor for CHD severity; the younger the patient, the lower the likelihood of myocardial infarction.

**Conclusion:**

ED severity and the time from ED to CHD onset may be predictive of coronary heart disease severity. Reducing smoking and alcohol consumption, maintaining a healthy body weight, and regular physical activity are important in preventing CVD in ED patients.

## 1 Introduction

Erectile Dysfunction (ED), which refers to the inability of men to consistently obtain and maintain a sufficient penile erection to accomplish a satisfactory sexual life, is a common sexual dysfunction that is closely related to men's physical and mental health and significantly affects the quality of life of patients and their partners (1). With the improvement of people's quality of life and the progress of social concepts, ED has been increasingly emphasized by men. However, due to multiple factors such as staying up late, lack of exercise, and obesity, the incidence of ED is increasing, and the prevalence rate in young men is as high as 30% (2–4). Coronary heart disease (CHD), which is known as coronary atherosclerotic heart disease and includes stable angina (SA), unstable angina (UA), and myocardial infarction (MI), is the most common type of cardiovascular disease (CVD) (5).

Decreased penile erectile function is considered an early manifestation of systemic atherosclerosis and an early sign of CVD (6). Furthermore, CVD is a predictor and risk factor for ED. Circulatory diseases may also affect the penile arteries, so patients with CVD may also develop ED (7), a case-control study in China found that CHD may increase the risk of developing ED (8) and suggested that approximately 44% to 75% of CVD patients suffer from varying degrees of ED (8). A recent meta-analysis showed that the RR of CVD events in ED patients was 1.47 (7), suggesting that cardiovascular events are more likely to occur in ED patients compared to the normal population. One study cost-analyzed ED as a marker for CVD diagnosis and intervention, suggesting that screening men with ED for CVD significantly reduces the burden on society and can be used for secondary prevention of CVD (9).

Previous studies have found a significant association between ED patients and subsequent angina, myocardial infarction, and stroke (10). Epidemiologic investigations have found common risk factors and pathogeneses between ED and CVD (11). Age, smoking, body mass index (BMI), total cholesterol (TC), triglycerides (TG), and diabetes mellitus, are common risk factors for both. Impairment of vascular endothelial function is thought to be a common pathogenesis of ED and CVD (12), and long-term administration of PDE-5 inhibitors increases the number of circulating endothelial progenitor cells and up-regulates cell-expressed NO synthase, which improves vascular endothelial function and reduces cardiovascular risk in patients with ED (13, 14). The idea that ED is a predictor of CVD is well recognized, and the Princeton III Consensus (expert panel) pointed out that exercise stress testing (EST), carotid intima-media thickness (CIMT), and noninvasive assessment of endothelial function are effective methods for the assessment of cardiovascular risk in individuals (15), but there are few studies related to observing the severity of ED and the clinical features of CHD. How does ED predict CVD? We hypothesized that the severity of ED is significantly associated with the clinical features of CHD, and more severe ED patients are more likely to develop serious CHD. Therefore, this study was designed in the hope that it could provide new evidence for the prevention of CVD risk in ED patients.

## 2 Materials and methods

### 2.1 Source of cases

A total of 248 patients aged from 18 to 65 years old who were admitted to the cardiac diagnosis and treatment center ward of the First Affiliated Hospital of Henan University of Chinese Medicine between November 2022 and May 2023 were screened as part of a cross-sectional study. All of these patients had a history of ED before the diagnosis of CHD. Patients with the following conditions were mainly excluded: (1) patients who were unconscious or had severe communication disorders; (2) patients without regular sexual partners (fixed sexual partners can effectively control the bias of patients' ED data); and (3) patients with concomitant localized lesions affecting erectile function, such as sclerodactyly and anatomical malformations. This study was approved by the Ethics Review Committee of the First Affiliated Hospital of Henan University of Chinese Medicine (project approval number: 2023HL-122).

### 2.2 Diagnostic criteria

CHD diagnostic criteria refer to coronary angiography results of ≥50% stenosis. The CHD subgroup diagnostic criteria were as follows: SA refers to the 2007 edition of the Diagnostic and Treatment Guidelines for Chronic Stable Angina (16); UA refers to the Chinese Society of Cardiovascular Disease in Medicine, Recommendations for Diagnosis and Treatment of Unstable Angina (17); MI (old, acute) refers to the 2019 edition of the Guidelines for Rapid Diagnosis and Treatment of Acute Coronary Syndrome Emergency (18). ED diagnostic criteria refer to the AUA Guidelines for the Diagnosis and Treatment of Erectile Dysfunction (19): those who are unable to achieve or maintain an erection of the penis sufficient for the completion of consoling sexual intercourse and for whom the course of the disease had lasted more than 3 months. Disease grading criteria: according to the International Erectile Function Rating Scale (IIEF-5), the degree of ED condition was categorized as mild, moderate, or severe.

### 2.3 Contents of the questionnaire

First, informed consent was obtained and all patients included in the study signed an informed consent form. We used a face-to-face questionnaire, with some information coming from patients' self-reports (e.g., exercise level, smoking history, etc.) and some from patients' electronic records (e.g., coronary angiography results). Detailed recordings were made of the patients' general information (age, BMI, physical activity, smoking, history of alcohol consumption), underlying disease information (hypertension, diabetes, hyperlipidemia, etc.), specifics of CHD (disease staging, lesion location, degree of stenosis, etc.), psychological status (GAD-7 and PHQ-9 scales), and erectile function (IIEF-5 scores, duration of ED, etc.). The patients were also asked to measure their height, weight, and blood pressure and undergo blood tests and urinalysis to assess liver and kidney function, blood lipids, blood glucose, and so on.

### 2.4 Quality control

The investigators involved in the survey were trained to maintain the confidentiality of the personal information of the patients involved in this study and the content of the survey, and after the collection of information from the clinical case questionnaire had been completed, Excel software was used to establish a database and double entry was made to check the salient values and to check for omissions and deficiencies.

### 2.5 Statistical analysis

SPSS 26. 0 statistical software was used, and the mean ± standard deviation ($\bar{x}\pm s$) was used to express the measurements that conformed to the normal distribution, and the count data were expressed as frequency (%). In this study, *χ*^2^ test was used for frequency distribution; some continuous variables were converted to ordered categorical variables using quartile and equal spacing methods; multiple sets of data were analyzed using one-way ANOVA; K-means clustering was used to classify patients with different clinical characteristics; independent risk factors for the degree of ED condition were determined using multifactorial ordered logistic regression analysis for variables that were statistically significant by one-way ordered logistic test and those that might be biologically related based on experience; and the independent risk factors for each studied factor were calculated as the odds ratio (OR) and its 95% confidence interval (95% CI). A *P*-value of <0.05 was considered to be statistically significant.

## 3 Results

### 3.1 Basic patient information

A total of 267 questionnaires were distributed and 248 valid questionnaires were returned, with a validity rate of 93%. The mean age of patients included in the study was 52.7 ± 9.12 years; mean body mass index (BMI) was 25.98 ± 3.3; mean duration of ED was 9.19 ± 12.18 months before symptoms of CHD; and mean duration of CHD was 16 ± 24.59 months. All patients had a history of ED prior to the diagnosis of CHD.

### 3.2 Comparison of patients’ clinical characteristics

In the comparison of general data, age, literacy level, and weekly exercise, the *χ*^2^ test showed statistical differences associated with the degree of ED condition. In terms of age, patients ≤40 years old had predominantly mild ED, and patients >50 years old had predominantly severe ED. In terms of literacy, patients with primary and secondary education had predominantly moderate to severe ED, and patients with university education had predominantly mild to moderate ED. In terms of the amount of weekly exercise, patients with <4 h of exercise had predominantly moderate to severe ED, and those with >4 h of exercise had predominantly mild ED, as shown in Table 1.

**Table 1:** Descriptive statistics of included data for CHD patients according to ED subtype (*N* = 248) [cases (%)].

Item and classification		Cases	ED subgroup			*P* value
			Mild	Moderate	Severe	
General information						
Age	≤40	28	21 (75.0)	2 (7.1)	5 (17.9)	*P *< 0.05
	41–50	59	30 (50.8)	18 (30.5)	11 (18.6)	
	>50	161	34 (21.1)	59 (36.6)	68 (42.2)	
BMI	<24	73	28 (38.4)	20 (27.4)	25 (34.2)	*P *= 0.57
	≥24	175	57 (32.6)	59 (33.7)	59 (33.7)	
Job type	Non-manual labor	73	32 (43.8)	20 (27.4)	21 (28.8)	*P *= 0.10
	Semi-manual labor	91	33 (36.3)	26 (28.6)	32 (35.2)	
	Manual labor	84	20 (23.8)	33 (39.3)	31 (36.9)	
Degree of education	Middle and primary school	106	23 (21.7)	43 (40.6)	40 (37.7)	*P *< 0.05
	Senior high school	83	33 (39.8)	21 (25.3)	29 (34.9)	
	University and above	59	29 (49.2)	15 (25.4)	15 (25.4)	
Weekly exercise	<4 h	221	70 (31.7)	76 (34.4)	75 (33.9)	*P *< 0.05
	>4 h	27	15 (55.6)	3 (11.1)	9 (33.3)	
Smoking	Yes	163	48 (29.4)	55 (33.7)	60 (36.8)	*P *= 0.09
	No	85	37 (43.5)	24 (28.2)	24 (28.2)	
Alcoholism	Yes	120	36 (30.0)	39 (32.5)	45 (37.5)	*P *= 0.34
	No	128	49 (38.3)	40 (31.3)	39 (30.5)	
Underlying disease information						
No. of underlying diseases	≤2	125	65 (52.0)	43 (34.4)	17 (13.6)	*P *< 0.05
	≥3	123	20 (16.3)	36 (29.3)	67 (54.5)	
Hypertension	Yes	163	40 (24.5)	59 (36.2)	64 (39.3)	*P *< 0.05
	No	85	45 (52.9)	20 (23.5)	20 (23.5)	
Diabetes	Yes	86	19 (22.1)	24 (27.9)	43 (50.0)	*P *< 0.05
	No	162	66 (40.7)	55 (34.0)	41 (25.3)	
Hyperlipidemia	Yes	87	20 (23.0)	32 (36.8)	35 (40.2)	*P *< 0.05
	No	161	65 (40.4)	47 (29.2)	49 (30.4)	
Anxiety state	Yes	94	15 (16.0)	20 (21.3)	59 (62.8)	*P *< 0.05
	No	154	70 (45.5)	59 (38.3)	25 (16.2)	
Depressive state	Yes	98	20 (20.4)	20 (20.4)	58 (59.2)	*P *< 0.05
	No	150	65 (43.3)	59 (39.3)	26 (17.3)	
Coronary heart disease data						
CHD subgroup	SA	86	45 (52.3)	33 (38.4)	8 (9.3)	*P *< 0.05
	UA	74	18 (24.3)	32 (43.2)	24 (32.4)	
	MI	88	22 (25.0)	14 (15.9)	52 (59.1)	
Diseased region	Anterior descending branch	222	66 (29.7)	73 (32.9)	83 (37.4)	*P *< 0.05
	No	26	19 (73.1)	6 (23.1)	1 (3.8)	
	Circumflex artery	124	25 (20.2)	37 (29.8)	62 (50.0)	*P *< 0.05
	No	124	60 (48.4)	42 (33.9)	22 (17.7)	
	Right coronary artery	122	28 (23.0)	31 (25.4)	63 (51.6)	*P *< 0.05
	No	126	57 (45.2)	48 (38.1)	21 (16.7)	
No. of stenotic vessels	1	95	50 (52.6)	36 (37.9)	9 (9.5)	*P *< 0.05
	2–3	91	28 (30.8)	30 (33.0)	33 (36.3)	
	>3	62	7 (11.3)	13 (21.0)	42 (67.7)	
Degree of stenosis	50%–70%	94	45 (47.9)	37 (39.4)	12 (12.8)	*P *< 0.05
	≥70	154	40 (26.0)	42 (27.3)	72 (46.8)	
Interventional therapy	No	95	45 (47.4)	35 (36.8)	15 (15.8)	*P *< 0.05
	Balloon dilatation	45	18 (40.0)	12 (26.7)	15 (33.3)	
	Stent implantation	108	22 (20.4)	32 (29.6)	54 (50.0)	

Data are presented as number (percentage) of patients. BMI, body mass index; ED, erectile dysfunction; CHD, coronary heart disease; SA, stable angina; UA, unstable angina; MI, myocardial infarction.

In the comparison of underlying disease conditions, the number of underlying diseases and the presence or absence of an underlying disease *χ*^2^ test were statistically different and associated with the degree of ED condition. In terms of the number of underlying diseases, patients with ≤2 underlying diseases were dominated by mild to moderate ED, and patients with ≥3 underlying diseases were dominated by moderate to severe ED. In terms of hypertension, diabetes mellitus, hyperlipidemia, anxiety status, and depression status, patients with the presence of the above diseases were dominated by moderate to severe ED, and patients without the presence of the above diseases were dominated by mild to moderate ED; the specifics of these diseases are shown in Table 1.

In the comparison of CHD information, there were associations between the ED subgroup and the CHD subgroup, lesion site, number of stenotic vessels, degree of stenosis, and mode of interventional intervention according to the *χ*^2^ test. In terms of CHD subtypes, patients with mild-to-moderate ED were more likely to present with SA, and patients with moderate-to-severe ED were more likely to present with UA and MI. In terms of the number of stenotic vessels, patients with mild-to-moderate ED were more likely to present with a single stenosis, and patients with moderate-to-severe ED more often had multiple vessel stenosis. In terms of the degree of stenosis, patients with mild-to-moderate ED had less severe stenosis, and patients with moderate-to-severe ED had more severe stenosis, as shown in Table 1.

### 3.3 Comparison of time from ED to CHD onset with disease information

**Figure 1:**
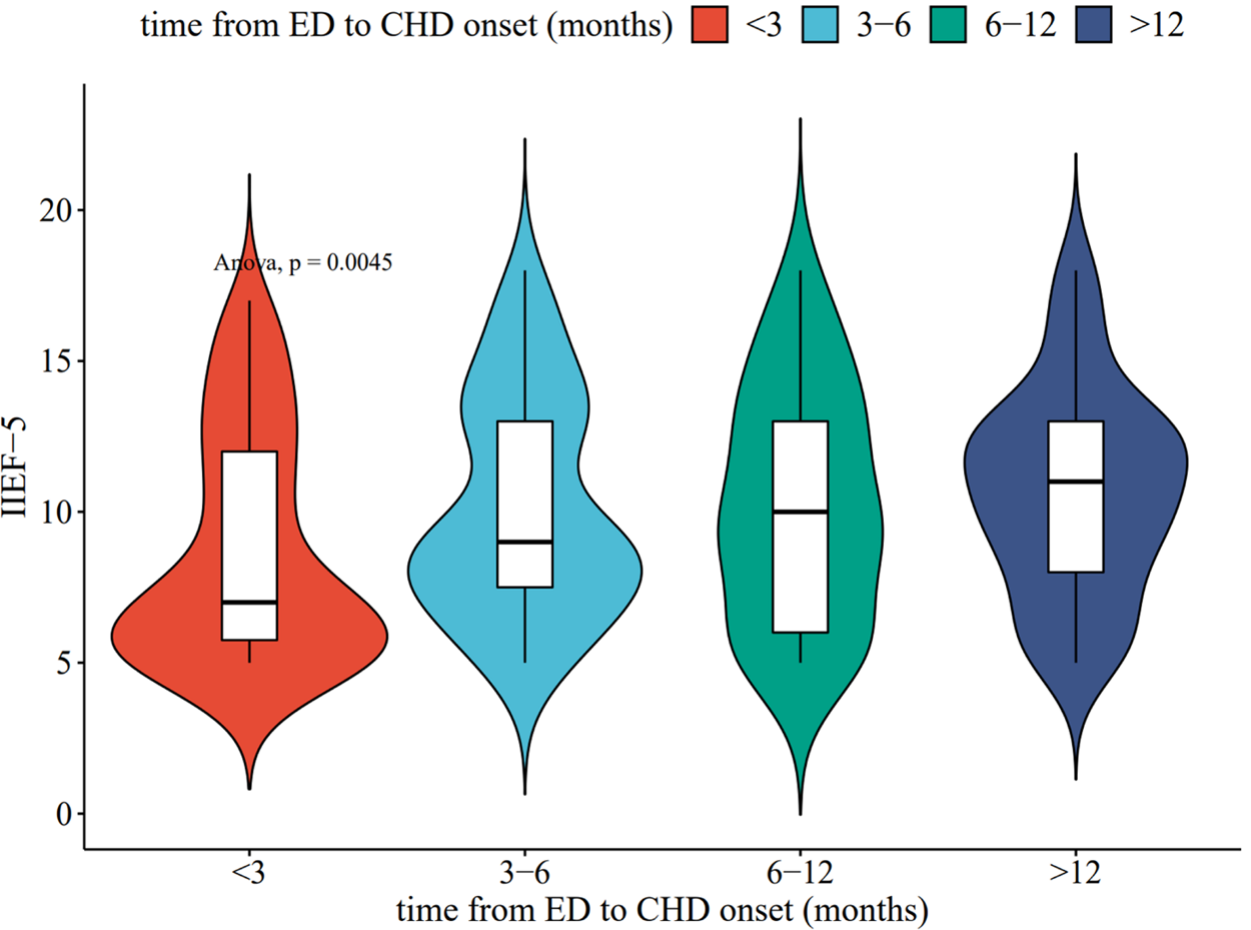
The time from ED to CHD onset and IIEF-5 association violin plot (*P* value < 0.05).

Recent meta-analysis (20) showed that shorter duration of ED plays a key role in the development of CVD and CHD. Therefore, we determined the time of the patient's first ED occurrence as well as the time of diagnosis of CHD, thus obtaining the time from ED to CHD onset. In terms of CHD subgroups, SA was predominant when the CHD had a longer time from ED onset (>12 months), and MI was predominant when the time to onset was shorter (<3 months). In terms of the number of stenotic vessels, the number of stenoses was predominantly 1 when the CHD was at a longer time from the onset of ED (>12 months), and more than 3 when the onset was shorter (<3 months). Then, we plotted the time from ED to CHD onset with IIEF-5 scores in violin plots; one-way ANOVA suggested *P* < 0.05; and the time from ED to CHD onset was positively associated with patients' IIEF-5 scores in all cases. This suggests that the IIEF-5 score may be an expressive factor of the time from ED to CHD onset. The more severe the ED, the earlier the onset of CHD may be, as shown in Table 2 or Figure 1.

**Table 2:** Descriptive statistics of CHD patients’ disease profile according to the time from ED to CHD onset (*N* = 248) [cases (%)].

Item and classification		Cases	Time from ED to CHD onset (months)				*P* value
			<3	3–6	6–12	>12	
CHD subgroup	SA	86	18 (20.9)	10 (11.6)	20 (23.3)	38 (44.2)	*P *< 0.05
	UA	74	19 (25.7)	28 (37.8)	12 (16.2)	15 (20.3)	
	MI	88	47 (53.4)	17 (19.3)	5 (5.7)	19 (21.6)	
No. of stenotic vessels	1	95	19 (20.0)	18 (18.9)	18 (18.9)	40 (42.1)	*P *< 0.05
	2–3	91	30 (33.0)	30 (33.0)	14 (15.4)	17 (18.7)	
	>3	62	35 (56.5)	7 (11.3)	5 (8.1)	15 (24.2)	

Data are presented as number (percentage) of patients. ED, erectile dysfunction; CHD, coronary heart disease; SA, stable angina; UA, unstable angina; MI, myocardial infarction.

### 3.4 K-means clustering of clinical characteristics of patients

We performed K-means clustering on the main clinical characteristics of patients, divided into “low-risk group” and “high-risk group” for clustering, and used one-way ANOVA to test the variability, and the results showed that the patients in the low-risk group had a low degree of ED, a low number of underlying diseases, a long time from ED to CHD onset, a low degree of CHD, a low number of stenotic vessels, and a low degree of stenosis, while the patients in the high-risk group had a high degree of ED, a high number of underlying diseases, a short time from ED to CHD onset, a heavy degree of CHD, and a large number of stenotic vessels with a high degree of stenosis, as shown in Table 3.

**Table 3:** Clinical characteristics of patients K-means clustering results (*N* = 248).

Clinical characteristics of patients	Cluster		*P* value
	Low risk group (*n* = 144)	High risk group (*n* = 104)	
Zscore: ED subgroup	−0.47367	0.65585	*P *< 0.05
Zscore: Number	−0.57275	0.79304	*P *< 0.05
Zscore: CHD subgroup	−0.49779	0.68925	*P *< 0.05
Zscore: No. of stenotic vessels	−0.59051	0.81762	*P *< 0.05
Zscore: Degree of stenosis	−0.4774	0.66102	*P *< 0.05
Zscore: Time from ED to CHD onset (months)	0.2588	−0.35834	*P *< 0.05

ED, erectile dysfunction; CHD, coronary heart disease.

### 3.5 Ordered logistic regression analysis of CHD disease degree

CHD was categorized according to stable angina, unstable angina, and myocardial infarction, and one-way ordered logistic regression analysis was performed on the data of general information and underlying disease information, and it was found that BMI, smoking, alcoholism, the number of underlying diseases, diabetes, anxiety state, and depressive state were risk factors for the severity of CHD (*P* < 0.05). The higher the value of these factors, the more severe the degree of CHD. Age is a protective factor for the severity of CHD; the younger the patient, the lower the likelihood of MI. Furthermore, all relevant factors were included in a multifactorial ordered logistic regression analysis (parallel line test: *P* = 0.133), and it was found that BMI, smoking, diabetes, and anxiety state were independent risk factors for CHD severity (*P* < 0.05) and age was an independent protective factor for CHD severity (*P* < 0.05), as shown in Table 4.

**Table 4:** Results of ordered logistic regression of risk factors for degree of CHD condition (*N* = 248).

Item and classification	Parallel line test	B	SE	Wald*χ*^2^	*OR value*	95% *CI*	*P* value
Results of one-factor ordered logistic regression							
Age	*P *= 0.374	−1.461	0.4313	11.477	0.232	(0.10, 0.54)	*P *< 0.05
BMI	*P *= 0.204	0.686	0.2643	6.739	1.986	(1.183, 3.335)	*P *< 0.05
Smoking	*P *= 0.234	1.109	0.2542	19.041	3.032	(1.842, 4.991)	*P *< 0.05
Alcoholism	*P *= 0.695	0.793	0.2383	11.090	2.211	(1.386, 3.527)	*P *< 0.05
No. of underlying diseases	*P *= 0.703	0.437	0.0891	24.098	1.548	(1.30, 1.844)	*P *< 0.05
Diabetes	*P *= 0.241	1.219	0.2606	21.879	3.383	(2.03, 5.637)	*P *< 0.05
Anxiety state	*P *= 0.792	1.352	0.2554	28.047	3.867	2.344, 6.378)	*P *< 0.05
Depressive state	*P *= 0.532	1.196	0.2496	22.959	3.307	2.027, 5.393)	*P *< 0.05
Multifactor ordered logistic regression results							
Age	*P *= 0.133	−1.532	0.4665	10.779	0.216	(0.087, 0.539)	*P *< 0.05
BMI	*P *= 0.133	0.964	0.2973	10.518	2.623	(1.465, 4.698)	*P *< 0.05
Smoking	*P *= 0.133	0.837	0.3415	6.011	2.310	(1.183, 4.512)	*P *< 0.05
Alcoholism	*P *= 0.133	0.323	0.3281	0.970	1.381	(0.726, 2.628)	*P *= 0.33
No. of underlying diseases	*P *= 0.133	−0.266	0.1597	2.783	0.766	(0.560, 1.048)	*P *= 0.10
Diabetes	*P *= 0.133	0.927	0.3387	7.494	2.527	(1.301, 4.908)	*P *< 0.05
Anxiety state	*P *= 0.133	1.025	0.3732	7.538	2.786	(1.341, 5.789)	*P *< 0.05
Depressive state	*P *= 0.133	1.103	0.3586	9.461	0.766	(0.56, 1.048)	*P *= 0.10

Collinearity statistics: VIF, variance inflation factor value <10; BMI, body mass index.

## 4 Discussion

With the improvement of social living standards and the impact of the rising incidence of ED in recent years, ED has been increasingly emphasized by men. Penile erection is the result of the synergistic cooperation of multiple factors, and any abnormality in any aspect will lead to ED, but its essence is still vascular nerve activity. Current research suggests that the common pathogenesis of ED and CVD may be based on endothelial dysfunction. Impaired endothelial function leads to decreased vasoconstriction and diastolic function, which is the main cause of vascular ED, while ED may be the first symptom of endothelial injury and may be followed by CVD. Previous studies have focused on verifying the conclusion that ED is a predictor of CVD, especially CHD as a disease. In the present study, we enrolled 248 patients who presented with CHD after ED to explore the relationship between CHD and both ED severity of disease and time from ED to CHD onset in an attempt to further reveal the intrinsic link between ED and CVD.

The results of the study found that in the comparison of general information, age, education, and weekly exercise were associated with the distribution of ED severity; in the comparison of underlying disease information, the number of underlying diseases, hypertension, diabetes, hyperlipidemia, anxiety state, and depression were associated with the distribution of ED severity; and in the comparison of CHD disease information, the degree of ED severity associated with the subgroups of CHD, lesion sites, number of stenoses, degree of stenosis, and interventional interventions. In addition, the time from ED to CHD onset was equally associated with IIEF-5 score, subgroups of CHD, and number of stenotic vessels. IIEF-5 score may be the expression factor of time from ED to CHD onset. The more severe the ED, the earlier the CHD may occur. Finally, in ordered logistic regression analysis, we found that BMI, smoking, diabetes, and anxiety state were independent risk factors for CHD severity (*P* < 0.05), and age was an independent protective factor for CHD severity.

In terms of age, erectile function declines with men's age, a finding confirmed in recent studies (21, 22), and the mechanism may be related to the decline in testosterone levels and the large deposition of collagen fibers in the penile corpus cavernosum (23); with age, vascular endothelial growth factor (VEGF) becomes markedly under-signaled, leading to a decline in vascular endothelial function, an obvious trigger for coronary heart disease (24, 25). VEGF plays a role in the development of vasogenic erectile dysfunction, and patients with the presence of VEGF disorders have a significant decrease in erectile function compared to the normal population (26, 27). In terms of literacy, patients with higher education have a milder degree of ED condition: higher education is an indicator of higher socio-economic status, and more literate ED patients pay more attention to ED treatment and maintain good lifestyle habits (28). In terms of amount of exercise, patients with weekly fitness habits are more likely to have mild ED, which is consistent with recent reports (29). Exercise improves the quality of arterial ED by decreasing endothelial cell apoptosis (30), so maintaining good exercise habits has a therapeutic effect on ED and early CVD (31). Smoking, alcoholism, hypertension, diabetes mellitus, and hyperlipidemia are all risk factors for ED and CHD. The pathogenesis is related to a decrease in vasodilatory factors (nitric oxide, hydrogen sulfide) and an increase in vasoconstrictive factors (angiotensin II, endothelin 1, and aldosterone) (32), which involves various pathways, such as oxidative stress and endothelial damage, neurovascular damage and repair, and endocrine dysfunction (33, 34). With regard to anxiety and depressive states, the degree of ED increases with the degree of anxiety and depression in patients; anxiety and depressive states can significantly lead to psychogenic ED, and brain network analysis in this group of patients shows obvious abnormalities (35). Based on the high prevalence of anxiety and depression in Chinese ED patients, Chinese clinicians should pay more attention to the early diagnosis and treatment of psychiatric symptoms in ED patients (36).

Through K-means clustering, it was found that patients with fewer underlying diseases had milder symptoms at the time of ED and did not develop CHD immediately after ED, and after CHD, these patients had fewer stenotic vessels and less severe stenosis, which more often manifested as SA. Patients with more underlying diseases had more severe symptoms at the time of ED and developed CHD soon after ED, and after CHD, the stenosis was more frequent and more severe, which more often manifested as MI. This provides a guideline for predicting the risk of CHD in patients with ED. Studies have reported that different types of CAD are associated with significant differences in peri-coronary fat and plaque characteristics (37, 38), and dyslipidemia is likewise a major risk factor for ED patients. Patients with dyslipidemia are at increased risk of ED, and statins have a protective effect and are a key factor in the secondary prevention of cardiovascular events (39, 40). This suggests that lipid status may be the main reason for the existence of an association between ED and CHD.

We believe that patients with mild ED conditions have a slow decline in sexual function, suggesting that lipid parameters are in a state of slow alteration (41), and endothelial dysfunction may also occur but progresses slowly; therefore, CAD occurs at a slower rate. At this time, PDE-5 inhibitors show improved function on endothelial cells, and early administration can effectively exert an ameliorative effect on ED and CVD risk (13). In patients with severe ED conditions, especially those with a rapid decline in sexual function in the short term, which suggests that lipid parameters are in a state of rapid change, endothelial dysfunction to atherosclerosis occurs rapidly, the trend is more acute, and the likelihood of CAD in a short period of time is also greater. Currently, it is recommended that patients undergo early CVD-related examinations; the use of statin interventions can effectively reduce the risk of CVD, and at the same time, ED and have a therapeutic effect on ED at the same time (42). Guidelines recommend that exercise stress testing (EST), carotid intima-media thickness (CIMT), and noninvasive assessment of endothelial function are effective methods for assessing individual cardiovascular risk after ED (15). Although all ED patients should undergo vascular screening, especially for ED of vascular origin, the urgent question is how to assess or grade the CVD risk of ED patients for further customized treatment options (43, 44).

Our study reveals that different levels of ED have the potential to predict the severity of CVD, and guidelines (45) recommend that SCORE2 or SCORE2-OP (Systematic Coronary Risk Estimation 2 and Systematic Coronary Risk Estimation 2-Older Persons) can be used as a tool to assess the risk of CVD in ED patients, which will have a positive impact on the prevention of CVD in ED patients. In addition, the risk factors for CHD severity suggest that reducing smoking and alcohol consumption, maintaining a healthy body weight, and regular physical activity are important in preventing CVD in ED patients. Currently, this study still has some limitations: (1) the patients in this study were collected from a single institution with a limited sample size. In the future, we will conduct this study in multiple medical institutions to increase the sample size; (2) the time from ED to CHD onset was determined based on the patients' self-reported history, which is highly subjective. In the future, we will use a more objective assessment by following up with ED patients.

## Data Availability

The original contributions presented in the study are included in the article/Supplementary Material, further inquiries can be directed to the corresponding author.
