# Supplementary material for: Does erectile dysfunction predict cardiovascular risk? A cross-sectional study of clinical characteristics in patients with erectile dysfunction combined with coronary heart disease
Source: Front Cardiovasc Med. 2024 Mar 18;11:1341819. doi: 10.3389/fcvm.2024.1341819 (PMC10984325; doi:10.3389/fcvm.2024.1341819)
Supplement: Supplementary file 1 [file Table1.docx]

Table 1 Basic information of 248 patients with ED combined with CHD

|  | Age | BMI | IIEF-5 | Duration of ED (months) | Duration of CHD (months) |
| --- | --- | --- | --- | --- | --- |
| Mean | 52.70 | 25.98 | 9.79 | 9.19 | 16.00 |
| Standard deviation | 9.12 | 3.30 | 3.70 | 12.18 | 24.59 |
| Minimum | 27 | 18.62 | 5 | 1 | 1 |
| maximum | 65 | 41.52 | 18 | 112 | 136 |

Table 2 Comparison of general data of 248 patients with ED combined with CHD [cases (%)]

| Item and classification | | Case | ED subgroup | | | *P value* |
| --- | --- | --- | --- | --- | --- | --- |
|  |  |  | Mild | Moderate | Severe |  |
| Age | ≤40 | 28 | 21(75.0) | 2(7.1) | 5(17.9) | *P*<0.001 |
|  | 41~50 | 59 | 30(50.8) | 18(30.5) | 11(18.6) |  |
|  | >50 | 161 | 34(21.1) | 59(36.6) | 68(42.2) |  |
| BMI | <24 | 73 | 28(38.4) | 20(27.4) | 25(34.2) | *P*=0.572 |
|  | ≥24 | 175 | 57(32.6) | 59(33.7) | 59(33.7) |  |
| Residence | City | 145 | 58(40.0) | 36(24.8) | 51(35.2) | *P*=0.012 |
|  | Country | 103 | 27(26.2) | 43(41.7) | 33(32.0) |  |
| Job type | Brain | 73 | 32(43.8) | 20(27.4) | 21(28.8) | *P*=0.099 |
|  | Semi-manual | 91 | 33(36.3) | 26(28.6) | 32(35.2) |  |
|  | Manual labour | 84 | 20(23.8) | 33(39.3) | 31(36.9) |  |
| Degree of education | Middle and primary school | 106 | 23(21.7) | 43(40.6) | 40(37.7) | *P*=0.003 |
|  | Senior high school | 83 | 33(39.8) | 21(25.3) | 29(34.9) |  |
|  | University and above | 59 | 29(49.2) | 15(25.4) | 15(25.4) |  |
| Dietary habit | Light | 89 | 36(40.4) | 25(28.1) | 28(31.5) | *P*=0.319 |
|  | Spicy or Greasy | 82 | 25(30.5) | 32(39.0) | 25(30.5) |  |
|  | Spicy and Greasy | 77 | 24(31.2) | 22(28.6) | 31(40.3) |  |
| Sleep quality | Preferably | 122 | 45(36.9) | 37(30.3) | 40(32.8) | *P*=0.709 |
|  | Bad | 126 | 40(31.7) | 42(33.3) | 44(34.9) |  |
| Weekly exercise | <4 hours | 221 | 70(31.7) | 76(34.4) | 75(33.9) | *P*=0.015 |
|  | >4 hours | 27 | 15(55.6) | 3(11.1) | 9(33.3) |  |
| Chainsmoker | Yes | 163 | 48(29.4) | 55(33.7) | 60(36.8) | *P*=0.09 |
|  | No | 85 | 37(43.5) | 24(28.2) | 24(28.2) |  |
| Tippler | Yes | 120 | 36(30.0) | 39(32.5) | 45(37.5) | *P*=0.341 |
|  | No | 128 | 49(38.3) | 40(31.3) | 39(30.5) |  |

Table 3 Comparison of underlying disease information in 248 patients with ED combined with CHD [cases (%)]

| Item and classification | | Case | ED subgroup | | | *P value* |
| --- | --- | --- | --- | --- | --- | --- |
|  |  |  | Mild | Moderate | Severe |  |
| No. of underlying diseases | ≤2 | 125 | 65(52.0) | 43(34.4) | 17(13.6) | *P*<0.001 |
|  | ≥3 | 123 | 20(16.3) | 36(29.3) | 67(54.5) |  |
| Hypertension | Yes | 163 | 40(24.5) | 59(36.2) | 64(39.3) | *P*<0.001 |
|  | No | 85 | 45(52.9) | 20(23.5) | 20(23.5) |  |
| Diabetes | Yes | 86 | 19(22.1) | 24(27.9) | 43(50.0) | *P*<0.001 |
|  | No | 162 | 66(40.7) | 55(34.0) | 41(25.3) |  |
| Hyperlipidaemia | Yes | 87 | 20(23.0) | 32(36.8) | 35(40.2) | *P*=0.021 |
|  | No | 161 | 65(40.4) | 47(29.2) | 49(30.4) |  |
| Anxiety state | Yes | 94 | 15(16.0) | 20(21.3) | 59(62.8) | *P*<0.001 |
|  | No | 154 | 70(45.5) | 59(38.3) | 25(16.2) |  |
| Depressive state | Yes | 98 | 20(20.4) | 20(20.4) | 58(59.2) | *P*<0.001 |
|  | No | 150 | 65(43.3) | 59(39.3) | 26(17.3) |  |

Table 4 Comparison of disease profiles in 248 patients with ED combined with CHD [cases (%)]

| Item and classification | | Case | ED subgroup | | | *P value* |
| --- | --- | --- | --- | --- | --- | --- |
|  |  |  | Mild | Moderate | Severe |  |
| CHD subgroup | SA | 86 | 45(52.3) | 33(38.4) | 8(9.3) | *P*<0.001 |
|  | UA | 74 | 18(24.3) | 32(43.2) | 24(32.4) |  |
|  | MI | 88 | 22(25.0) | 14(15.9) | 52(59.1) |  |
| Diseased region | Anterior descending branch | 222 | 66(29.7) | 73(32.9) | 83(37.4) | *P*<0.001 |
|  | No | 26 | 19(73.1) | 6(23.1) | 1(3.8) |  |
|  | Circumflex artery | 124 | 25(20.2) | 37(29.8) | 62(50.0) | *P*<0.001 |
|  | No | 124 | 60(48.4) | 42(33.9) | 22(17.7) |  |
|  | Right coronary artery | 122 | 28(23.0) | 31(25.4) | 63(51.6) | *P*<0.001 |
|  | No | 126 | 57(45.2) | 48(38.1） | 21(16.7) |  |
| No. of stenotic vessels | 1 | 95 | 50(52.6) | 36(37.9) | 9(9.5) | *P*<0.001 |
|  | 2~3 | 91 | 28(30.8) | 30(33.0） | 33(36.3） |  |
|  | >3 | 62 | 7(11.3) | 13(21.0) | 42(67.7) |  |
| Degree of stenosis | 50%~70% | 94 | 45(47.9) | 37(39.4) | 12(12.8) | *P*<0.001 |
|  | ≥70 | 154 | 40(26.0) | 42(27.3) | 72(46.8) |  |
| Interventional therapy | No | 95 | 45(47.4) | 35(36.8) | 15(15.8) | *P*<0.001 |
|  | Balloon dilatation | 45 | 18(40.0) | 12(26.7) | 15(33.3) |  |
|  | Stent implantation | 108 | 22(20.4) | 32(29.6) | 54(50.0) |  |

Table 5 Comparison of time between ED and CHD onset and disease information in 248 patients with ED combined with CHD [cases (%)]

| Item and classification | | Case | CHD time from ED onset (month) | | | | *P value* |
| --- | --- | --- | --- | --- | --- | --- | --- |
|  |  |  | <3 | 3-6 | 6-12 | >12 |  |
| CHD subgroup | SA | 86 | 18(20.9) | 10(11.6) | 20(23.3) | 38(44.2) | *P*<0.001 |
|  | UA | 74 | 19(25.7) | 28(37.8) | 12(16.2) | 15(20.3) |  |
|  | MI | 88 | 47(53.4) | 17(19.3) | 5(5.7) | 19(21.6) |  |
| No. of stenotic vessels | 1 | 95 | 19(20.0) | 18(18.9) | 18(18.9) | 40(42.1) | *P*<0.001 |
|  | 2~3 | 91 | 30(33.0) | 30(33.0） | 14(15.4） | 17(18.7) |  |
|  | >3 | 62 | 35(56.5) | 7(11.3) | 5(8.1) | 15(24.2) |  |

Table 6：The results of Pearson correlation coefficient test between ED severity level and included information

General information

|  | Age | BMI | Residence | Job type | Education | Dietary habit | Sleep quality | Exercise | Chainsmoker | Tippler |
| --- | --- | --- | --- | --- | --- | --- | --- | --- | --- | --- |
| r value | .340^**^ | 0.114 | 0.064 | .136^*^ | -.192^**^ | 0.089 | 0.044 | -0.092 | .130^*^ | 0.093 |
| p value | 0.000 | 0.074 | 0.319 | 0.033 | 0.002 | 0.160 | 0.490 | 0.147 | 0.040 | 0.146 |

Underlying disease information

|  | No. of underlying diseases | Hypertension | Diabetes | Hyperlipidaemia | Anxiety state | Depressive state |
| --- | --- | --- | --- | --- | --- | --- |
| r value | .504^**^ | .254^**^ | .250^**^ | .157^*^ | .447^**^ | .384^**^ |
| p value | 0.000 | 0.000 | 0.000 | 0.013 | 0.000 | 0.000 |

Coronary heart disease information

|  | CHD subgroup | No. of stenotic vessels | Degree of stenosis | Interventional therapy | time from ED to CHD onset (month) |
| --- | --- | --- | --- | --- | --- |
| r value | .391^**^ | .472^**^ | .328^**^ | .336^**^ | -.242^**^ |
| p value | 0.000 | 0.000 | 0.000 | 0.000 | 0.000 |

Note: **. Correlation is significant at the 0.01 level; *. Significant at the 0.05 level.

Table 7: Clinical characteristics of patients K-means final clustering results

| Clinical characteristics of patients | Cluster | | *P* value |
| --- | --- | --- | --- |
|  | Low risk group (n=144) | High risk group (n=104) |  |
| Zscore: ED subgroup | -0.47367 | 0.65585 | *P*<0.001 |
| Zscore: Number | -0.57275 | 0.79304 | *P*<0.001 |
| Zscore: CHD subgroup | -0.49779 | 0.68925 | *P*<0.001 |
| Zscore: No. of stenotic vessels | -0.59051 | 0.81762 | *P*<0.001 |
| Zscore: Degree of stenosis | -0.47740 | 0.66102 | *P*<0.001 |
| Zscore: Time from ED to CHD onset (month) | 0.25880 | -0.35834 | *P*<0.001 |

Table 8: Results of ordered logistic regression of risk factors for degree of ED condition

| Item and classification | B | SE | Waldχ2 | *OR value* | 95%*CI* | *P value* |
| --- | --- | --- | --- | --- | --- | --- |
| Results of one-factor ordered logistic regression | | | | | | |
| Age | 1.145 | 0.197 | 33.668 | 3.143 | (2.134, 4.627) | *P*<0.001 |
| Type of work | 0.313 | 0.148 | 4.458 | 1.368 | (1.022, 1.829) | *P*=0.035 |
| Degree of education | -0.452 | 0.150 | 9.035 | 0.637 | (0.474, 0.855) | *P*=0.003 |
| Smoking history | 0.515 | 0.248 | 4.306 | 1.674 | (1.029, 2.721) | *P*=0.038 |
| No. of underlying diseases | 0.787 | 0.101 | 61.091 | 2.197 | (1.804, 2.678) | *P*<0.001 |
| Hypertension | 1.043 | 0.255 | 16.664 | 2.837 | (1.719, 4.679) | *P*<0.001 |
| Diabetes | 1.003 | 0.254 | 15.620 | 2.725 | (1.657, 4.482) | *P*<0.001 |
| Hyperlipidaemia | 0.601 | 0.247 | 5.903 | 1.824 | (1.123, 2.962) | *P*=0.015 |
| Anxiety state | 1.912 | 0.271 | 49.641 | 6.765 | (3.975, 11.519) | *P*<0.001 |
| Depressive state | 1.588 | 0.259 | 37.455 | 4.893 | (2.942, 8.134) | *P*<0.001 |
| Multifactor ordered logistic regression results (parallel line test: *P*=0.054) | | | | | | |
| Age | 1.127 | 0.228 | 24.386 | 3.085 | (1.972, 4.826) | *P*<0.001 |
| Type of work | -0.013 | 0.285 | 0.002 | 0.987 | (0.565, 1.725) | *P*=0.963 |
| Degree of education | -0.342 | 0.298 | 1.319 | 0.710 | (0.396, 1.274) | *P*=0.251 |
| Smoking history | -0.032 | 0.290 | 0.012 | 0.968 | (0.549, 1.709) | *P*=0.912 |
| No. of underlying diseases | 0.169 | 0.236 | 0.509 | 1.184 | (0.745, 1.879) | *P*=0.476 |
| Hypertension | 0.454 | 0.383 | 1.406 | 1.574 | (0.744, 3.330) | *P*=0.236 |
| Diabetes | 0.314 | 0.355 | 0.782 | 1.369 | (0.682, 2.746) | *P*=0.377 |
| Hyperlipidaemia | 0.614 | 0.335 | 3.357 | 1.847 | (0.958, 3.561) | *P*=0.067 |
| Anxiety state | 1.225 | 0.428 | 8.201 | 3.405 | (1.473, 7.877) | *P*=0.004 |
| Depressive state | 0.716 | 0.394 | 3.296 | 2.045 | (0.945, 4.428) | *P*=0.069 |
